# Pancreaticoduodenectomy for pancreatic head cancer with cavernous transformation of the portal vein: a case report

**DOI:** 10.1186/s40792-022-01463-0

**Published:** 2022-06-30

**Authors:** Naohiro Hirano, Masahiro Iseki, Takanori Morikawa, Yuuichiro Umino, Shuichi Aoki, Koetsu Inoue, Shun Nakayama, Takayuki Miura, Kunihiro Masuda, Masaharu Ishida, Hideo Ohtsuka, Masamichi Mizuma, Kei Nakagawa, Kiyoshi Kume, Atsushi Masamune, Takashi Kamei, Michiaki Unno

**Affiliations:** 1 Department of Surgery, Tohoku University Graduate School of Medicine, 1-1 Seryo-machi, Aoba-ku, Miyagi 980-8574 Sendai, Japan; 2 Department of Gastroenterology, Tohoku University Graduate School of Medicine, 1-1 Seiryo-machi, Aoba-ku, Sendai, Miyagi 980-8574 Japan

**Keywords:** Cavernous transformation, Extrahepatic portal vein obstruction, Pancreatic cancer, Pancreaticoduodenectomy

## Abstract

**Background:**

Cavernous transformation of the portal vein (CTPV) due to extrahepatic portal vein obstruction is a rare vascular anomaly. Since its symptoms usually appear in childhood, most of the adult cases are detected unexpectedly with other diseases. Only a few reports have described surgical difficulties in patients with CTPV. We report a case of pancreatic head cancer with CTPV in a patient who underwent pancreaticoduodenectomy.

**Case presentation:**

A 77-year-old man with epigastric and back pain was referred to our hospital. Computed tomography revealed a tumor in the pancreatic head and a CTPV near the hepatic hilum. CTPV consisted of two main collateral vessels connected by multiple surrounding small vessels. Also, portal vein obstruction was observed near the hepatic hilum, which was far from the pancreatic head tumor. After confirming that there was no distant metastasis by a thorough whole-body search, we performed a pancreaticoduodenectomy following neoadjuvant chemotherapy. During the operation, we carefully manipulated the area of the CTPV and omitted lymph node dissection in the hepatoduodenal ligament to prevent massive venous bleeding and intestinal congestion. Pancreaticoduodenectomy was performed without any intraoperative complications and the postoperative course was uneventful. Complete tumor resection was histologically confirmed.

**Conclusion:**

Although pancreaticoduodenectomy for patients with CTPV involves many surgical difficulties, we successfully performed it by determining specific treatment strategies tailored to the patient and following careful and delicate surgical procedures.

## Background

Cavernous transformation of the portal vein (CTPV) is an uncommon vascular variant arising from extrahepatic portal vein obstruction (EHPVO) [1]. Multiple small collateral vessels appear around the portal vein (PV). Most cases of CTPV appear in childhood with symptoms of portal hypertension; the main clinical presentations are gastroesophageal variceal bleeding and hematologic abnormalities due to splenomegaly [2]. In adults, it is often found incidentally during examinations for other diseases. The discovery rate of CTPV is approximately one in 2000 using ultrasound (US) imaging screening for the abdomen, with an incidence of 15.6% among patients with EHPVO [3, 4]. Recently, CTPV are being detected more frequently with improvements in radiological imaging [4]. Surgical procedures can be complicated in patients with CTPV because of the irregular anatomy of the vessels and their fragility. Moreover, since pancreaticoduodenectomy for patients with pancreatic head cancer requires lymph node dissection around the pancreatic head, dissection of each small vessel related to CTPV may increase the risk of life-threatening intraoperative venous bleeding as well as intestinal congestion [5].

Pancreatic ductal adenocarcinoma (PDAC) is one of the most lethal malignancies, with a 5-year overall survival rate of < 10% [6, 7]. The incidence of PDAC has increased gradually over the past few decades. Although multidisciplinary treatment strategies, including adjuvant and neoadjuvant therapies, have played important roles in patients with PDAC, complete tumor resection remains a central component of PDAC treatment [6, 8].

Here, we report that pancreaticoduodenectomy was safely performed for complete tumor resection in a patient who had pancreatic head adenocarcinoma with CTPV. This is the first report that the patient whose CTPV was unrelated to the infiltration from PDAC, successfully underwent pancreaticoduodenectomy. The success was due to an accurate evaluation of resectability through various procedures and the omission of hepatoduodenal ligament lymph node dissection.

## Case presentation

A 77-year-old man presented to our hospital with back and epigastric pain. His past medical history included spinal canal stenosis. At the previous hospital, computed tomography (CT) revealed a tumor in the pancreatic head close to the PV. Additionally, PV stenosis was observed near the hepatic hilum, which was not the location of the tumor and serpiginous collateral vessels appeared around the extrahepatic bile duct. Pancreatic head cancer was suspected, along with a vascular anomaly. The patient was referred to our hospital for further evaluation and surgical treatment.

The patient did not have jaundice or symptomatic cholangitis. A blood test revealed that the blood count, biochemical examination, and tumor markers including carcinoembryonic antigen (CEA) and DUPAN-2, were within the normal range, while carbohydrate antigen 19-9 (CA19-9), SPan-1, and carbohydrate antigen 125 were elevated (208 U/ml, 54.8 U/ml, and 45.9 U/ml, respectively). No diabetes mellitus was detected in blood tests.

**Fig. 1:**
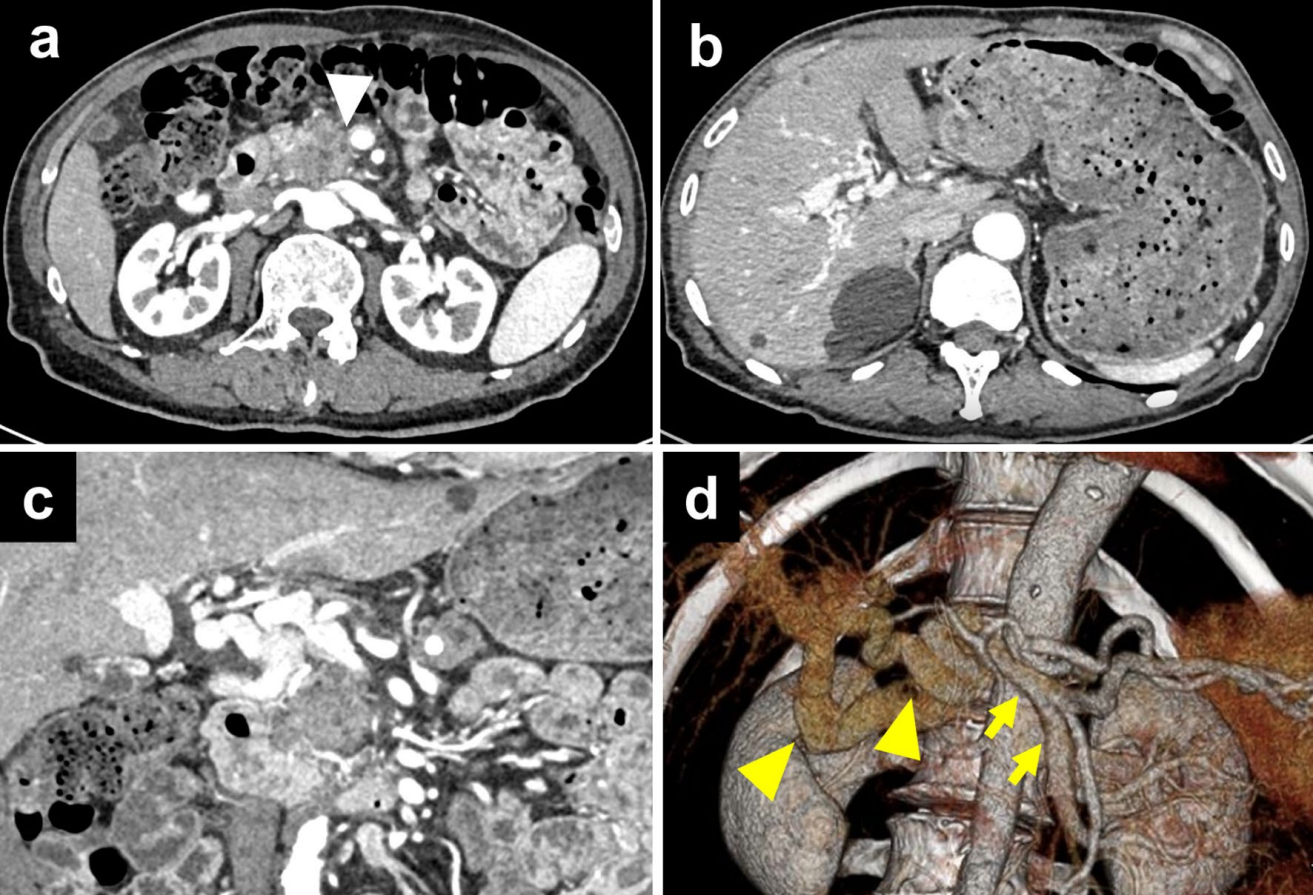
**a** An enhanced CT indicated a tumor abutted on the superior mesenteric vein in the level of maximum short-axis diameter (white arrowhead). **b** Axial view of the CT examination revealed cavernous transformation around the hepatic hilum. **c** Cranial view of the CT showed serpiginous collateral vessels leading to the intrahepatic portal vein. **d** Three-dimensional reconstruction image of the CT examination showed that two main collateral vessels (yellow arrowheads) existed around the hepatic portal region, and the superior mesenteric vein, the splenic vein and the portal vein in the pancreas had no sign of tumor invasion (yellow arrows)

**Fig. 2:**
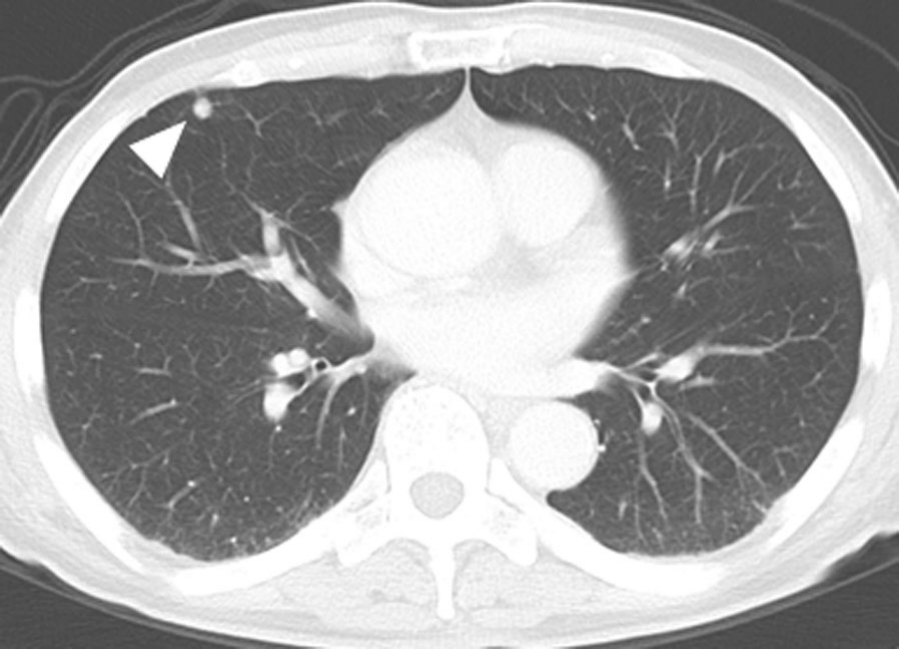
Computed tomography of the lung revealed a 6-mm-size nodule (white arrowhead) in the right middle lobe

**Fig. 3:**
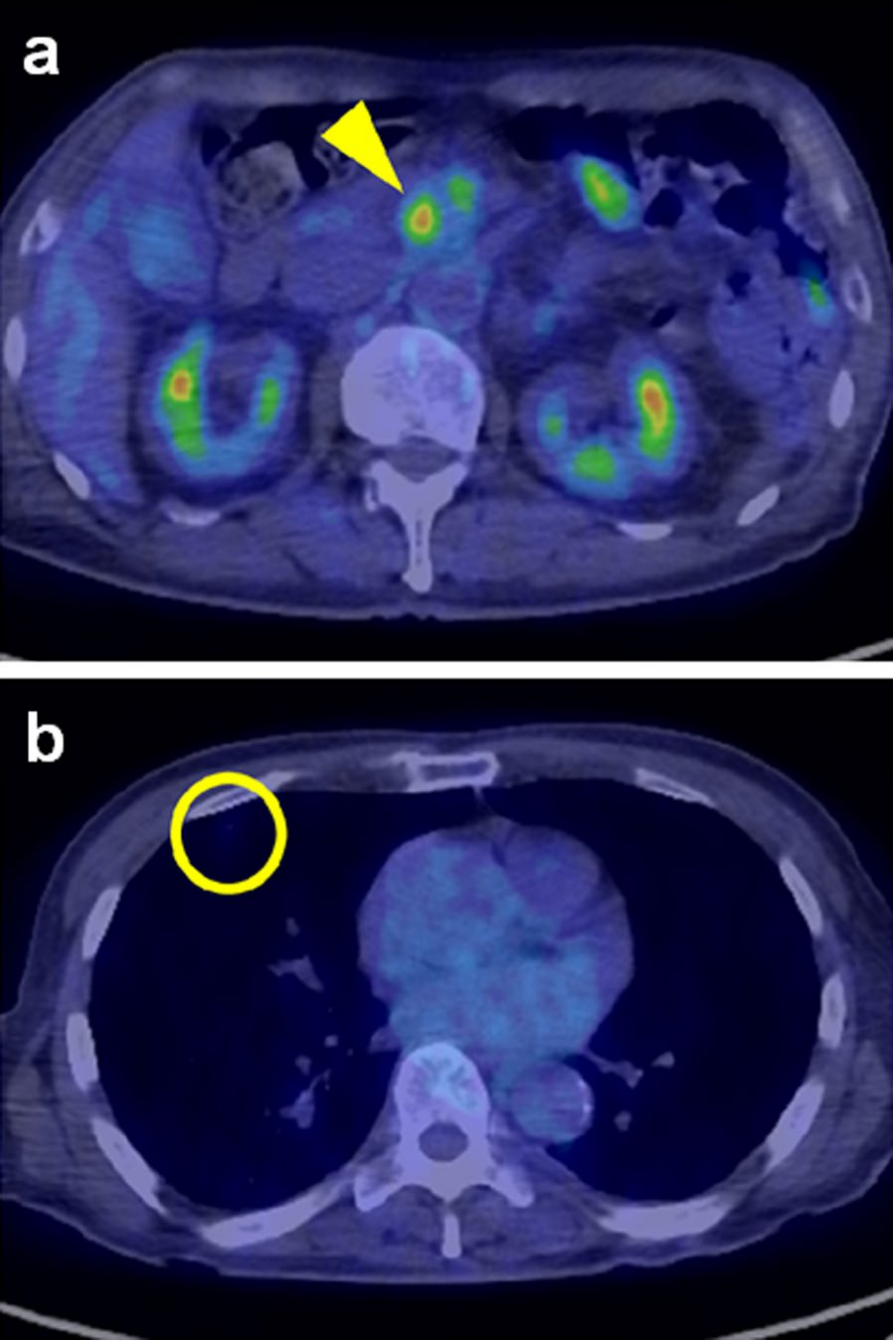
**a** In ^18^F fluoro-2-deoxyglucose positron emission tomography scan, the tumor in the pancreatic head was depicted with the maximum standardized uptake value of 6.46 (yellow arrowhead). **b** There was no obvious accumulation in the nodule of the right lung (yellow circle)

An enhanced CT examination performed at our hospital revealed that the tumor in the pancreatic head was close to the PV without apparent direct invasion and the CTPV, which included two main collateral vessels and small vessels, existing around the hepatic hilum (Fig. 1). The portal vein was obstructed in the hepatoduodenal ligament, where it was clearly separated from the tumor. Also, the CT images showed no signs of liver cirrhosis or portal hypertension in the superior mesenteric vein (SMV) and splenic vein (SV). Hemangiomas and cysts were detected in the liver. There was no evidence of hepatic metastasis in the magnetic resonance imaging (MRI). Although a lung nodule of 6 mm in the middle right lobe was detected in the CT images, no other distant metastasis was found (Fig. 2). Preoperative ^18^F fluoro-2-deoxyglucose positron emission tomography (FDG-PET) revealed that the tumor in the pancreatic head had a maximum standardized uptake value of 6.46. Meanwhile, there was no marked uptake in the nodule in the right lung or any other body area (Fig. 3).

A histological examination using endoscopic ultrasound-guided fine-needle aspiration confirmed that the tumor was an adenocarcinoma. Staging laparoscopy was performed to determine the presence of micrometastasis in the abdominal cavity and value lavage cytology. As a result, no metastasis to the liver or in the peritoneal cavity was revealed. The intraperitoneal lavage cytology was negative. This patient was diagnosed with resectable PDAC based on these results, and we administered gemcitabine and S-1 for 6 weeks (two cycles) as neoadjuvant chemotherapy. The planned chemotherapy cycles were completed without any major adverse events, and the size of the pancreatic head cancer was slightly reduced (30 to 28 mm). However, the nodule in the right lung did not change in size. In the blood test, CA19-9 and Span-1 significantly declined (16.8 and 12.4 U/ml, respectively). To exclude the possibility that the lung nodule was a metastasis, we performed resection of the lung nodule prior to pancreatectomy. Lung tumor was histologically identified as nodule derived from pneumoconiosis. After neoadjuvant chemotherapy and confirmation that the pancreatic head cancer was resectable without any distant metastasis, we decided that pancreaticoduodenectomy for pancreatic head cancer was an appropriate treatment for the patient, despite the associated high risk of venous bleeding and intestinal congestion.

**Fig. 4:**
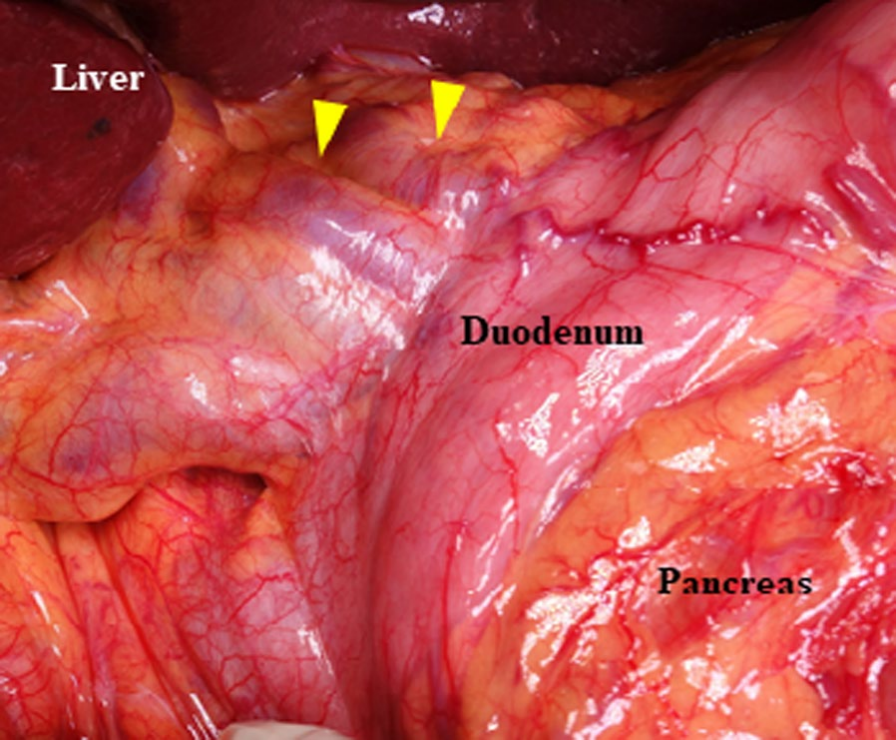
Intraoperative finding before the resection: blood vessels were extraordinarily developed in the hepatoduodenal mesentery. Two main collateral vessels (yellow arrowhead) and small vessels could be seen around there

**Fig. 5:**
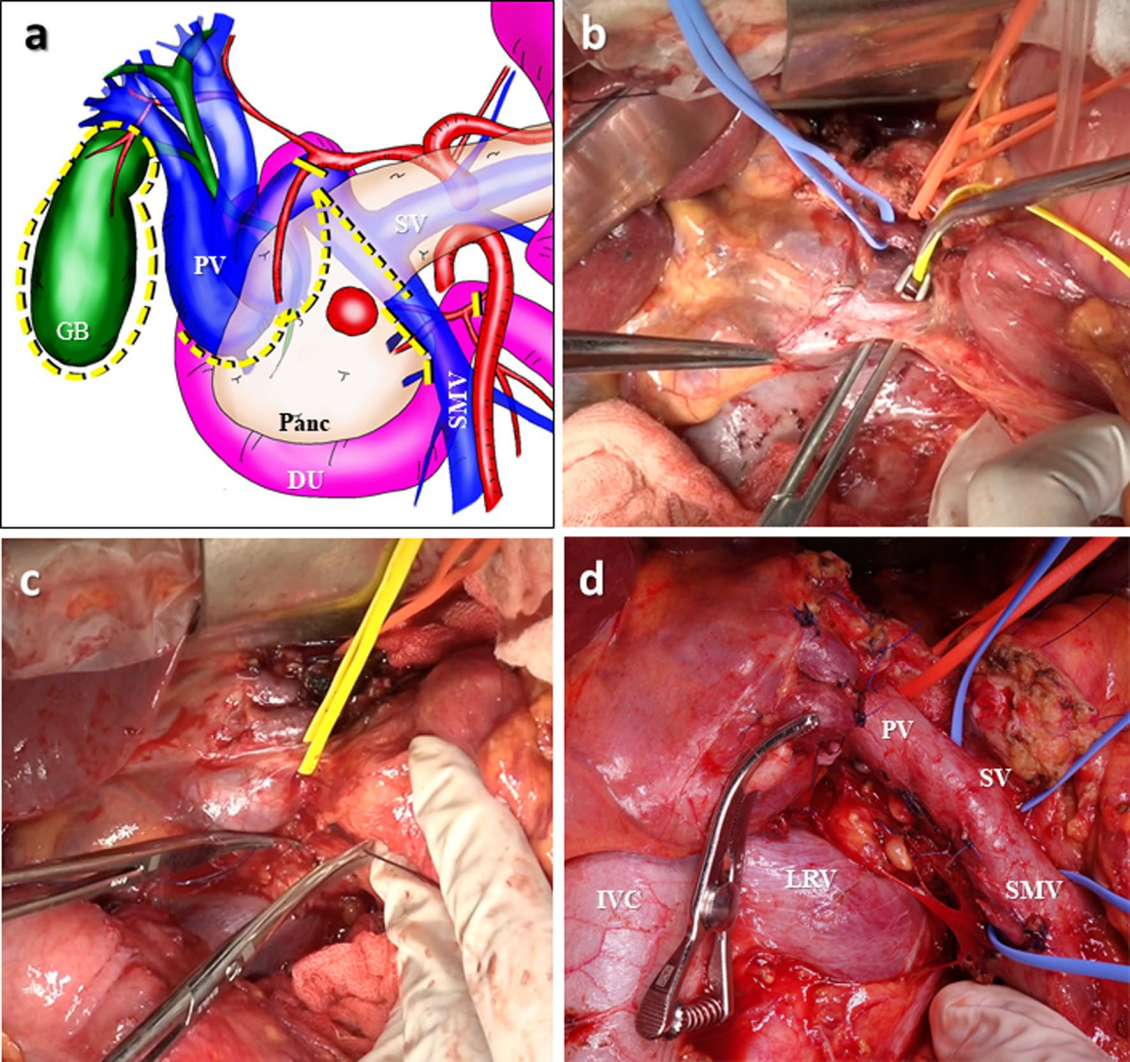
**a** Scheme of the resection line of the pancreaticoduodenectomy in this case (dotted line). **b** After dissecting the upper edge of the pancreas in the hepatoduodenal ligament, the common bile duct was encircled at the level of the upper edge of the pancreas. **c** The hepatoduodenal ligament including two major collateral vessels, was mostly preserved. **d** Intraoperative picture after pancreaticoduodenectomy: distal stump of the bile duct was clamped with a forceps. *GB* gall bladder, *DU* duodenum, *PV* portal vein, *SMV* superior mesenteric vein, *SV* splenic vein, *Panc* pancreas, *IVC* inferior vena cava, *LRV* left renal vein

Lymph node dissection in the hepatoduodenal ligament was preoperatively planned to be omitted due to the risk of venous bleeding and intestinal congestion. A bypass catheter made from heparinized hydrophilic polymer, the Anthron tube (Toray Industries, Tokyo, Japan), was also prepared for intestinal congestion associated with the transection of CTPV. The surgery was performed through a chevron incision. The CTPV was visible through the hepatoduodenal ligament (Fig. 4). The CTPV was only seen on the proximal side of the SV confluence, and dilation of the SV and SMV was not obvious. The blood pressure of the vessels in the CTPV area seemed high in accordance with the degree of bleeding; however, no other area had typical findings of portal hypertension. The most common bleeding site during surgery was the small blood vessels related to CTPV around the head of the pancreas. As a result, dissection of the pancreatic head along the PV was possible without massive bleeding. We dissected the upper edge of the pancreatic head and preserved major collateral vessels in the hepatoduodenal ligament. The bile duct was cut at the level of the common bile duct (i.e., the upper edge of the pancreatic head). Cholecystectomy was performed separately after the pancreaticoduodenectomy. CTPV also appeared around the gallbladder, and cholecystectomy required careful manipulation (Fig. 5). Reconstruction was routinely performed using a modified Child’s method, and the surgery resulted in no uncontrollable bleeding or venous intestinal congestion. We did not use the Anthron tube that had been prepared preoperatively during the operation. The surgical duration was 505 min, and the estimated intraoperative blood loss was 985 ml.

Microscopic examination of the pancreatic specimens confirmed that the tumor was completely resected with a sufficient surgical margin. Pathological findings also showed that the tumor was a mixed-type invasive ductal and anaplastic carcinoma. The tumor size was 18 × 16 × 15 mm and the total number of the dissected lymph nodes was fifty. Peri-pancreatic head lymph nodes and the peri-gastric lymph nodes included five metastases. Microscopically, lymphovascular and venous invasions were observed. In addition, the tumor invaded the anterior tissue of the pancreas. These were defined as T1N2M0, Stage III according to the 8th edition Tumor-Node-Metastasis staging system, which was developed by the Union for International Cancer Control. The patient was discharged on the 23rd postoperative day with an uneventful course.

Unfortunately, multiple liver metastases were observed in the CT 3 months after surgery. CTPV remained stable without new angiogenesis and disappearance in the hepatoduodenal ligament.

## Discussion

Pancreaticoduodenectomy for patients with CTPV is associated with life-threatening risks of intraoperative venous bleeding, and intestinal congestion due to the dissection of the vessels linked to the CTPV. In this case, pancreaticoduodenectomy was safely performed as a complete tumor resection for a patient with pancreatic head adenocarcinoma by preoperative intensive evaluation of resectability, intraoperative delicate manipulation, and partial omission of lymph node dissection.

EHPVO is recognized as a common cause of portal hypertension and is mainly attributed to congenital, intra-abdominal inflammatory, traumatic, neoplastic, or unknown causes [9–11]. Various methods, such as US, CT, and MRI, are available for the detection of CTPV due to EHPVO, and most adult cases are diagnosed accidentally. No universal theory that explains the morphology of the CTPV has been reported in the past because the developmental mechanism remains unclear. CTPV growing with a tumor, obstruction of the PV is mainly caused by direct compression by the tumor. In our case, the obstruction point was far from the location of the tumor. In addition, the patient had no history of inflammation, such as choledocholithiasis or pancreatitis. The cause of the obstruction was unclear; thus, this case was considered idiopathic EHPVO.

To the best of our knowledge, only a few surgical cases of CTPV have been reported, including laparoscopic left hepatectomy, laparoscopic cholecystectomy, distal gastrectomy, and pancreaticoduodenectomy [5, 11–13]. In the previously reported case of pancreaticoduodenectomy, EHPVO was caused by PV infiltration in pancreatic cancer [5]. This report showed that pancreaticoduodenectomy with PV resection could be accomplished using an SMV–PV bypass graft before tumor resection [5]. In our case, pancreaticoduodenectomy was safely performed without bypass, omitting lymph node dissection of the hepatoduodenal ligament and preserving collateral circulation.

Pancreatic cancer is refractory and requires multimodal therapy in combination with chemotherapy [6, 7]. Long-term survival and cure are reportedly possible only with complete tumor resection [14, 15]. Recently, improvements in surgical procedures and perioperative care have reduced the morbidity and mortality rates of pancreaticoduodenectomy, although they remain high in low-volume institutions [16, 17]. Regarding mortality rate after pancreaticoduodenectomy, age, complications, and body mass index (BMI) have been reported as patient-related risk factors [18]. Anatomical anomalies, including CTPV, are also considered to present a high surgical risk for pancreaticoduodenectomy. Regarding surgical treatment for CTPV, some previous reports recommended implementation by an experienced surgical team [13, 19]. They also mentioned that high-risk surgery, such as pancreaticoduodenectomy, should be optionally avoided or palliative surgery should be considered [13, 19]. Bockhorn et al. suggested that greater amounts of intraoperative blood transfusions are needed in patients with pancreatic diseases with CTPV [20]. Moreover, it is important to pay attention to liver ischemia or low perfusion caused by the dissection of vessels linked to CTPV, especially in patients who have already undergone multiple cycles of neoadjuvant hepatotoxic chemotherapy [5]. Since pancreaticoduodenectomy for a patient with CTPV has a tremendous surgical risk, it is important to decide the treatment modality rigorously and to plan the strategy for safe surgery.

Staging laparoscopy is the sole tool for the diagnosis of micrometastasis, which cannot be detected by radiological images [21, 22]. Since micrometastasis, such as positive peritoneal cytology, is a predictor of poor prognosis, staging laparoscopy is routinely performed at our institution in the case of radiologically observed resectable pancreatic cancer on preoperative images [21, 22]. We suggest that addressing poor prognostic factors prior to radical surgery is crucial, especially in high-risk cases such as the present one.

In this case, we omitted lymph node dissection of the hepatoduodenal ligament to safely perform surgery. Since lymph node dissection of the hepatoduodenal ligament was considered to contribute to a better prognosis than complete resection of the tumor without it, it is routinely performed for the patients with pancreatic head cancer in Japan. However, the optimal extent of lymph node dissection for PDAC still remains unclear. Imamura et al. reported that the rate of lymph node metastasis in the hepatoduodenal ligament of pancreatic head cancer is approximately 12.7% and prophylactic lymph node dissection in the hepatoduodenal ligament had limited effects on their survival [23]. Therefore, omission of lymph node dissection in the hepatoduodenal ligament may be acceptable in patients with high surgical risk, including the elderly, and patients with poor general conditions or anatomical anomalies, such as this case.

## Conclusions

Herein, we report a rare case of pancreatic head cancer with CTPV in a patient who underwent pancreaticoduodenectomy. No presence of distant metastasis was observed in preoperative examinations, including adequate preoperative imaging tests, staging laparoscopy, resection of the lung nodule, and pancreaticoduodenectomy was performed. Detailed perioperative strategies, such as omission of the lymph node dissection in hepatoduodenal ligament and careful surgical manipulations, ensured surgical safety, resulting in complete histological tumor resection.

## Data Availability

Not applicable.

## Abbreviations

CA19-9: Carbohydrate antigen 19–9
CEA: Carcinoembryonic antigen
CT: Computed tomography
CTPV: Cavernous transformation of the portal vein
EHPVO: Extra-hepatic portal vein obstruction
FDG-PET: ^18^F fluoro-2-deoxyglucose positron emission tomography
GDA: Gastroduodenal artery
MRI: Magnetic resonance image
PDAC: Pancreatic ductal adenocarcinoma
PV: Portal vein
SMV: Superior mesenteric vein
SV: Splenic vein
US: Ultrasonography
